# Measures of frequency and effect in clinical research

**DOI:** 10.1007/s11255-023-03626-w

**Published:** 2023-05-10

**Authors:** Graziella D’Arrigo, Mercedes Gori, Annalisa Pitino, Dimitrios G. Tsalikakis, Vassilios Liakopoulos, Stefanos Roumeliotis, Giovanni Tripepi

**Affiliations:** 1 Institute of Clinical Physiology (IFC), National Research Council (CNR), 89124 Reggio Calabria, Italy; 2 Institute of Clinical Physiology (IFC), National Research Council (CNR), 00100 Rome, Italy; 3 Department of Electrical and Computer Engineering, University of Western Macedonia, Kozani, Greece; 4 2nd Department of Nephrology, Medical School, AHEPA Hospital, Aristotle University of Thessaloniki, 54636 Thessaloniki, Greece; 5 Division of Nephrology and Hypertension, 1st Department of Internal Medicine, School of Medicine, AHEPA Hospital, Aristotle University of Thessaloniki, St. Kyriakidi 1, 54636 Thessaloniki, Greece

## Abstract

The assessment of risk and effect size of a specific endpoint associated to the presence/absence of a certain exposure is a hallmark in clinical and epidemiological research. In fact, before recommending any treatment, it is mandatory to investigate the magnitude of the benefits and harms between the exposure under investigation (e.g. a given treatment) and a specific disease or event. To do this, clinicians and statisticians use absolute (risk differences, number needed to treat, likelihood to be helped or harmed) and relative (risk ratio, incidence rate ratio, hazard ratio and odds ratio) measures of effect. Herein, using a series of clinical examples, we aim to present a step by step methodologic approach of measures of effect in the area of nephrology and urology.

## Introduction

The assessment of risk and effect size of a specific endpoint associated to the presence/absence of a certain exposure is a hallmark of clinical and epidemiological research. From a public health perspective, before recommending any treatment to modify the time course of a disease, it is mandatory to investigate the magnitude of the benefits and harms between the exposure under investigation (e.g. a given treatment) and a specific clinical condition or event. To do this, clinicians and statisticians use absolute [risk differences, number needed to treat (NNT), likelihood to be helped or harmed (LHH)] and relative (risk ratio, incidence rate ratio, hazard ratio and odds ratio) measures of effect [1]. Herein, by reporting a series of clinical examples, we aim to present a step by step methodologic approach aimed at clarifying the measures of effect in the area of nephrology and urology.

The risk or simply the cumulative incidence or incidence proportion (CIR), estimates the chances that an individual will experience an event or develop a disease during a specified period. It is calculated as number of patients with a new event occurring during a specific time interval divided by the total number of individuals at risk at the beginning of the observation. The risk ranges from 0 to 100%. Researchers can decide to use cumulative incidence to assess the risk of a disease or event over a short or long time periods. An example of cumulative incidence is the risk (“chance” or “likelihood”) of developing urolithiasis among people with high body mass index-BMI (≥ 30 kg/m^2^) values in a specific time window. CIR is also referred as “event rate”, however in randomized trials the CIR in the control group is often referred as control event rate (CER) or baseline risk, whereas the CIR of the treatment group is referred as experimental event rate (EER) or treatment event rate (TER).

To calculate the CIR of developing urolithiasis in obese and non-obese patients (BMI ≥ 30 kg/m^2^), we will use the following hypothetical data shown in Table 1.

**Table 1:** Association between urolithiasis and BMI

	Urolithiasis		Total
	Yes	No	
BMI ≥ 30 kg/m^2^	233 (a)	1886 (b)	2119
BMI < 30 kg/m^2^	236 (c)	3700 (d)	3936
Total	469	5586	6055

The risk (or CIR) of urolithiasis could be calculated for each group as follows:

Obese patients: CIR = *a*/*a* + *b* = 233/2119 = 0.11.

Non-obese patients: CIR = *c*/*c* + *d* = 236/3936 = 0.06.

## From the risk of disease to the measure of effect

The risks mentioned above, CIR, CER and EER can be compared by calculating the ratio or the difference of the two measures giving origin to two measures of effect:

1. the relative risk in RCTs, (odds ratio in case control studies)
2. the absolute risk quantifying the impact of the treatment

The absolute measures indicate the absolute effect of an intervention whereas the relative measures provide the strength of the association but do not allow an assessment of the absolute effect of an intervention, which depends, in addition, to the strength of the association. The absolute risk numbers are necessary to understand the implications of relative risks and how specific factors influence the likelihood of developing a disease or health condition.

## Absolute risk measurements

The absolute risk expresses the incidence of the disease among those exposed (or unexposed) to a risk factor, i.e. the proportion of individuals who develop the disease during the observation period. An absolute measure of the effect is the Risk Difference (RD), also known as the absolute risk reduction (ARR), that is useful to quantify the effect of an exposure on health of a population. This measure is derived from the difference between the risks of the two groups and it is useful to assess the impact of the removal of the exposure on the risk of a specific disease. If we consider the previous study in which we tested the association between a given exposure and the disease, the ARR is calculated as differences between the risks of exposed and unexposed groups:

$$ {\text{RD}} = {\text{ Risk of obese patients }} - {\text{ Risk of non-obese patients }} = 0.{11} - 0.0{6} = 0.0{5}. $$

This could be translated to clinicians and patients as follows: to be non-obese reduces the “absolute” risk of urolithiasis of 5% as compared with obese in a specific time period. The risk difference is naturally constrained (i.e., it is context specific), and this may generate problems when extending results to other patients. For example, if a given study estimates a risk difference of − 10% for a given endpoint, then for a group with an initial risk of 8%, the same endpoint will have an unplausible (i.e., negative) probability of − 2%. Such problems occurs when the results are extended to patients with different risks from those observed in the reference study. In clinical trials the risk difference can be also expressed in terms of risk increase (ARI). ARI can be used to specify the difference in terms of risk when the risk of outcome is increased by the experimental drug and it is calculated as the risk in treated minus the risk in untreated patients. Risk difference could be also called “attributable risk”, i.e. excess risk that can be attributed to be exposed. Moreover, in the context of a RCT, it is possible to calculate, also, the Relative Risk Reduction (RRR) as ratio between ARR and the risk in the control group:

$$ {\text{RRR}} = \, \left( {{\text{CIR}}_{{\text{control group}}} - {\text{ CIR}}_{{\text{treated group}}} } \right)/{\text{ CIR}}_{{\text{control group}}} = {\text{ARR}}/{\text{CIR}}_{{\text{control group}}} . $$

The RRR represents the proportion of the original baseline (or control) risk that is removed by treatment.

Other important measures used in RCT are the number needed to treat (NNT) and the number needed to harm (NNH). The NNT evaluates the benefits and risks of a given intervention, estimating the number of patients who need to be treated in order to gain a unit of benefit over the control treatment or how many patients we need to treat for one to be benefited. Thus, NNT is a measure of effect quantifying the exact number of patients that we need to treat in order to obtain a therapeutic benefit and so to prevent an event.

The NNT is the reciprocal of the absolute risk reduction and is necessary to be calculated in RCTs aiming to investigate the effect of a new treatment (for example a new drug agent) on a certain disease or condition.

$$ {\text{NNT }} = {\text{ 1/ARR}}. $$

Treatment efficacy increases at decreasing NNT, so 1 is the perfect NNT: one therapeutic success for each treated patient. NNT is very helpful to guide the decision-making process, so that the physician can make a better choice on whether or not to administer a certain therapy/drug. Given the properties of the NNT, between-trial or indirect comparisons based on the NNT of individual trials should be avoided when there are differences in baseline risk. NNT cannot be calculated when the risk difference is zero and presents some disadvantage. For example, it cannot be combined in a meta-analysis. A 95% confidence interval for the number needed to treat can be obtained simply by calculating reciprocals of the values defining the 95% confidence interval for the absolute risk reduction.

On the other hand, the NNH is the number of patients to be treated to observe an adverse effect of treatment. The NNH is the reciprocal of the absolute risk increase:

$$ {\text{NNH }} = { 1}/{\text{ARI}}. $$

To estimate the Risk-Efficacy balance of a treatment, starting from NNT and NNH, we use the likelihood of being helped or harmed (LHH), calculated as:

$$ {\text{LHH }} = \, \left( {{1}/{\text{NNT}}} \right)/\left( {{1}/{\text{NNH}}} \right). $$

LHH expresses the likelihood that a patient will benefit from the treatment versus stopping it. If LHH is greater than 1 the benefit of the drug overcome the risk, if it is equal to 1 the risks and benefits are identical, if it is less than 1 the risks overcome the benefits. In a hypothetical example, to investigate the renoprotective effect of a novel Sodium-glucose co-transporter-2 SGLT2 inhibitor, we designed a RCT enrolling 10.000 patients with CKD stages 1–3 and randomly divided them to the active or placebo group (5000 patients in each group). The endpoint was progression to end stage kidney disease (ESKD) in a follow-up period of 5 years. In the active group 700 patients developed ESKD and in the control group 900.

The risk in the active group is 700/5000 = 0.14 and in the control group 900/5000 = 0.18 and therefore the risk ratio is 0.14/0.18 = 0.78. This could be translated as follows: the novel drug offers a 22% risk reduction of the progression to ESKD. The absolute risk reduction on the other hand is 0.18–0.14 = 0.04 and from this measure by dividing 100/4 (or 1/0.04) we can calculate the NNT, which is 25. Therefore, we need to give the new SGLT2 inhibitor to 25 CKD patients for 5 years in order to avoid 1 patient progresses to ESKD. In the previous scenario, if the events that occurred (progression to ESKD) were ten times lower in each group (70 in the active and 90 in the placebo group), the risk in groups is 70/5000 = 0.014 in the active and 90/5000 = 0.018 in the placebo group and the risk ratio remains exactly the same 0.014/0.018 = 0.78. However, the absolute risk reduction is 0.018 – 0.014 = 0.004 and the NNT is 1000/4 = 250. Therefore, in the new scenario we need to treat 250 patients for 5 years to prevent one event. These examples show that the clinical impact of two drugs might be different, even if they have identical risk ratios.

## Relative risk measurements

### Odds ratio

An odds ratio (OR) is a measure of association representing the probability (odds) of an event occurring given an exposure to a risk factor, compared with the odds of the outcome occurring in the absence of that exposure.

While the odds ratio is most commonly used in case–control studies, the relative risk is used in prospective studies. The odds ratio measures the degree of correlation between two factors, for example between a disease (outcome) and a risk condition (exposure) and might reveal an exposure that could lead to a specific event.

Tips:

- do not confound odds with odds ratio
- odds and probability are different: odds is the ratio of the probability that the event of interest occurs to the probability that it does not
- odds ratio is not so intuitive as relative risk

The OR is a good estimate of the RR when a disease is rare (< 10%).

The OR is calculable in case–control studies whereas the RR in prospective studies.

We will use the previous example of a group of patients affected by urolithiasis in relation to their condition of obesity. From this example, we will calculate the odds ratio between observed frequencies in obese and in non-obese group. odds of urolithiasis (disease) in obese patients is: *a*/*b* = 233/1886 = 0.12od ds of urolithiasis (disease) in non-obese patients is: *c*/*d* = 236/3700 = 0.06

The odds ratio is 0.12/0.06 = 1.94. Odds of urolithiasis is 94% higher in obese compared to non-obese patients; considering that the 95% confidence interval goes from 1.6028 to 2.3406 and do not include 1 is statistically significant (*p* < 0.001).

## Measures of precision: the confidence interval

A confidence interval expresses the amount of potential variation in a point estimate (a measure that may represent the mean value, RR, OR etc.). This variation is attributable to the fact that our point estimate (of the mean value or hazard ratio etc.), is based on a sample of the population rather than the whole population.

From the above example (Table 1), we might conclude that patients with BMI ≥ 30 having an OR of 1.94 are about twice as likely to develop urolithiasis than those with BMI < 30 (OR = 1.94).

$$95\% \mathrm{CI}=\mathrm{exp}\left\{\mathrm{ln}(\mathrm{OR})\pm 1.96*\sqrt{\left[\frac{1}{a}+\frac{1}{b}+\frac{1}{c}+\frac{1}{d}\right]}\right\},$$

$${95\% \mathrm{CI}}_{\mathrm{low}}=\mathrm{exp}\left\{\mathrm{ln}\left(1.94\right)+1.96*\sqrt{\left[\frac{1}{233}+\frac{1}{1886}+\frac{1}{236}+\frac{1}{3700}\right]}\right\}=1.6028,$$

$${95\% \mathrm{CI}}_{\mathrm{high}}=\mathrm{exp}\left\{\mathrm{ln}\left(1.94\right)-1.96*\sqrt{\left[\frac{1}{233}+\frac{1}{1886}+\frac{1}{236}+\frac{1}{3700}\right]}\right\}=2.3406.$$

In a 95% confidence interval ranging from an OR = 1.60 to an OR = 2.34, an OR of 1.94, is the point estimate we get from this clinical study. It must be pointed out, however, that not all subjects with BMI ≥ 30 can have been included in our study, so that the efficacy estimate, 1.94, is based on a particular sample of people with high BMI (BMI ≥ 30). If we assume that we can take other samples of people from the same baseline population from which the patients with BMI ≥ 30 have been taken for our study, we would get more point estimates; not all of them exactly equal to 1.94. Some samples are likely to show efficacy below 1.94 and some above 1.94.

The 95% CI is a range that will contain the true (real population) value of the parameter, 95% of the time if we were to repeat the experiment/study several times. Thus, if we were to repeat the experiment/study, 95 intervals out of 100 would give an interval that contains the true OR above from us calculated. We can interpret the CI only in relation to repeated sampling.

This could be translated to the following: patients with BMI ≥ 30 having an OR of 1.94 are about twice as likely to develop urolithiasis than those with BMI < 30 (OR = 1.94), but we must remember that this measure could range from a low OR of 1.6 to a high OR of 2.3.

The confidence interval also provides information about the precision of an estimate. The narrower the confidence interval, the more precise the estimate. Generally, larger sample sizes provide a more precise estimate while estimates with wide confidence intervals should be interpreted with caution.

## Measures of significance: *p* value

The “*p*” value expresses the probability that the difference between the observed value and the null value (no effect) occurred by “chance” or, more specifically, occurred simply because of sampling variability. The smaller the “*p*” value, the lower the probability that the difference is due to chance. Generally, a “*p*” value less than 0.05 is used as a cut-off point (albeit an arbitrary one); it means that there is less than a 5% probability that the detected difference between the observed differences in measures (ORs, RRs etc.) are not real (significant) but rather due to sampling variability and thus due to chance. If the “*p*” value is less than 0.05, the observed differences between the various calculated measures are called “statistically significant.”

It is possible to calculate, also, the “odds of exposition” that matches with the above mentioned “odds of disease”: odds of obesity(exposition) in presence of urolithiasis so calculated: *a*/*c* = 233/236 = 0.987 odds of obesity (exposition) in absence of urolithiasis so calculated: *b*/*d* = 1886/3700 = 0.510

The odds ratio is 0.987/0.510 = 1.94 so that the odds of obesity is 94% higher in obese than in non-obese patients.

## How is the ODDS ratio interpreted?

An odds ratio may vary from zero to infinity.

If OR = 1 the odds of exposition is equal in presence and absence of disease. In this case exposition is irrelevant to the occurrence of the disease, so that there is not any association between exposition and disease.

If OR > 1 the odds of exposition is higher in presence than in absence of disease. In this case exposition is relevant to the occurrence of the disease, so that there is positive association between exposition and disease.

If OR < 1 the odds of exposition is lower in presence than in absence of disease. In this case exposition is protective to the occurrence of the disease, so that there is negative association between exposition and disease.

## Risk ratio

When we consider risk in a study it is possible to calculate the risk ratio, also called relative risk, that is a measure comparing the risk in two groups of patients. Risk ratio compares the risk of event (i.e. disease, cardiovascular event, death) in one group with a given risk in comparison with another group with different risk. Two groups are typically differentiated according to exposure (i.e. exposed to a certain risk factor, unexposed), or other factor (i.e. demographic characteristic such as male versus female). The primary interest group is referred to as the exposed group, while the comparison group is referred to as the unexposed group. Therefore, risk ratio is calculated as ratio between risk in exposed group and risk in unexposed group.

It is well known that lung cancer is the leading cause of mortality in the United States and the world. Although the association between tobacco smoking and lung cancer has been widely studied, an estimated 16,000 lung cancer deaths occur annually in Americans who have never smoked cigarettes. This relevant topic was further investigated in the National Institutes of Health-AARP (NIH-AARP) Diet and Health study [2]. The association between heavy drinking and lung cancer was re-examined in an selected sample including 8000 individuals (unpublished data, see Table 2).

**Table 2:** Association between heavy drinking and lung cancer

Drinking status	Lung cancer		Total
	Yes	No	
Heavy	66 (a)	3334 (b)	3400 (a + b)
Not heavy	54 (c)	4546 (d)	4600 (c + d)
Total	120 (a + c)	7880 (b + d)	8000 (N)

$$RR=\frac{a/(a+b)}{c/(c+d)}=\frac{66/3400}{54/4600}=1.65.$$

This result implies that heavy drinkers have a 65% higher risk, than not heavy, to develop cancer (*p* = 0.006).

The 95% confidence interval is:

$${95\mathrm{\% CI}}_{\mathrm{RR}}=\mathrm{exp}\left\{\mathrm{ln}\left(\mathrm{RR}\right)\pm 1.96*\sqrt{\left[\frac{b}{a*(a+b)}+\frac{d}{c*(c+d)}\right]}\right\},$$

$${95\% \mathrm{CI}}_{\mathrm{low}}=\mathrm{exp}\left\{\mathrm{ln}\left(\mathrm{RR}\right)-1.96*\sqrt{\left[\frac{3334}{66*3400}+\frac{4546}{54*4600}\right]}\right\}=1.16,$$

$${95\mathrm{\% CI}}_{\mathrm{low}}=\mathrm{exp}\left\{\mathrm{ln}\left(1.65\right)-1.96*\sqrt{\left[\frac{3334}{66*3400}+\frac{4546}{54*4600}\right]}\right\}=1.16,$$

$${95\mathrm{\% CI}}_{\mathrm{high}}=\mathrm{exp}\left\{\mathrm{ln}\left(1.65\right)+1.96*\sqrt{\left[\frac{3334}{66*3400}+\frac{4546}{54*4600}\right]}\right\}=2.36.$$

How is the risk ratio interpreted?

A risk ratio may vary from zero to infinity.

If RR = 1 the risk of the event in the two groups is the same, so that there is no association between the exposure and the outcome.

If RR > 1 means that the risk of the event in the treated or exposed group is higher than in the control group. In this case, the exposure is dangerous.

If RR < 1 means that the risk of the event in the treated or exposed group is lower than in the control group. In this case, the exposure is protective.

The RR shows the strength of the association between the exposure (or treatment) and the outcome.

According to the guidelines Consolidated Standards of Reporting Trials (CONSORT) [3] and Strengthening the Reporting of Observational Studies in Epidemiology 2007 (STROBE) [4] it is necessary to report both relative and absolute measurements of effect to provide a complete description of association.

Remember that, in order to provide a complete information about risks, you should use absolute and relative risk.

In fact, the relative risk of two studies could be the same but the absolute measurements could be different as in the following example (Table 3).

**Table 3:** Relative risk calculation in the two studies

Study	Absolute risk exposed	Absolute risk unexposed	Relative risk
1	0.38	0.13	3
2	0.05	0.02	3

## Conclusions

The assessment of the association between a given risk factor and a certain outcome represents a hallmark of epidemiological and clinical research. In clinical epidemiology, the research question dictates the choice of study design and this latter in turns largely affects the choice of the measures of effect, relative or absolute. Relative measures of effect (such as risk ratio, incidence rate ratio, hazard ratio, and odds ratio) have the advantage to provide the strength of the causal relationship between a given exposure and a specific endpoint and for this reason they are well suited in etiological research. Relative measures of effect per se are not sufficient to claim that a given exposure has prognostic value for a given endpoint, an issue which formally demands the assessment of the accuracy of the exposure being investigated as a predictor of the outcome. Vice-versa, absolute measures of risk have the advantage to provide the probability of a given event over a time period in exposed and unexposed individuals and for this reason they are well suited in prognostic research. The risk difference is commonly used in interventional studies to calculate the number needed to treat (NNT), i.e. the number of patients that we need to treat to prevent 1 adverse event in a given time period.

## Data Availability

All data used for this article are available.

## Abbreviations

CIR: Cumulative incidence or incidence proportion
CER: Control event rate
EER: Experimental event rate
TER: Treatment event rate
RCT: Randomised controlled trial
RD: Risk difference
ARR: Absolute risk reduction
ARI: Absolute risk increase
RR: Risk ratio
RRR: Relative risk reduction
NNT: Number needed to treat
NNH: The number needed to harm
OR: Odds ratio
CI: Confidence interval
